# Medium term results of total knee arthroplasty as a primary treatment for knee fractures

**DOI:** 10.1051/sicotj/2017060

**Published:** 2018-03-16

**Authors:** Ayman Ebied, Ahmed Zayda, Sameh Marei, Hany Elsayed

**Affiliations:** Menoufia University Hospitals, Shebin El Kom Egypt

**Keywords:** Total knee arthroplasty, Knee fractures, Osteoarthritis, Knee

## Abstract

*Introduction*: Successful treatment of knee comminuted periarticular fractures associated with osteoporosis and pre-existing arthritis is a challenging task.

*Methods*: This is a prospective study on 27 patients who had comminuted intra and periarticular knee fractures and pre-existing arthritis. Fractures were classified according to Muller's AO classification. Primary knee arthroplasty was performed ± internal fixation following 4 weeks of splinting. A stem was added to the tibial tray and Legacy Constrained Condylar Knee (LCCK) or Rotating Hinge (RH) prosthesis were used depending on the level of ligament damage and bone defects. The Knee Society Score (KSS) and radiological evaluation were performed at 3, 6 and 12 months then annually thereafter.

*Results*: The average age of this group of patients was 63 years (range 59–74). Sixteen knees received primary femoral component and Posterior Stabilized insert, while 8 had LCCK. RH implants were chosen in 2 and distal femoral replacement was necessary in one knee. Twenty five patients were available for the final review at an average 6 years in whom the KSS was 80 (range 75–89) points. All patients achieved full knee extension and average knee flexion of 110° (range 90–135°). One knee needed re-admission for early Debridement Antibiotic Irrigation and Retention (DAIR) but none of the knees was revised or awaiting revision.

*Conclusion*: Knee arthroplasty achieves highly successful outcome when performed as a primary treatment for comminuted intra and periarticular knee fractures in elderly patients. Survival of implants and functional range of movement at midterm are excellent.

## Introduction

The dilemma of treating elderly patients with intra or periarticular fractures in presence of knee arthritis is still a challenge to orthopedic surgeons. Internal fixation is usually difficult due to osteoporosis and metaphyseal comminution [1,2]. The elderly patients are usually difficult to mobilize with partial weight bearing strategy. Therefore, early weight bearing can cause failure of fixation. The alternative of non-weight bearing ± immobilization is associated with muscle wasting, joint stiffness and other general complications [3].

Failed fixation of knee peri-articular fractures can be salvaged by total knee arthroplasty (TKA). However, knee arthroplasty for non or malunited fractures is associated with higher incidence of complications when compared to TKA for arthritis. In one series the rate of intraoperative and postoperative complications reached 30% for each. Additionally the functional range of movement (ROM) and survivorship were inferior to the results of TKA for arthritis [4]. Notably, joint stiffness, infection and damage of the extensor mechanism were reported when total knee replacement was performed as a salvage procedure for complicated tibial plateau fractures [5,6].

There is paucity in the literature in regard to the use of primary TKA as a treatment for intra and peri articular fractures. Additionally, published series included small numbers of patients and only short term results were reported [7–9].

In this article results of total knee arthroplasty as a primary treatment for intra and peri-articular fractures of arthritic knees in elderly patients is reported at the medium term follow up.

## Material and methods

**Figure 1:**
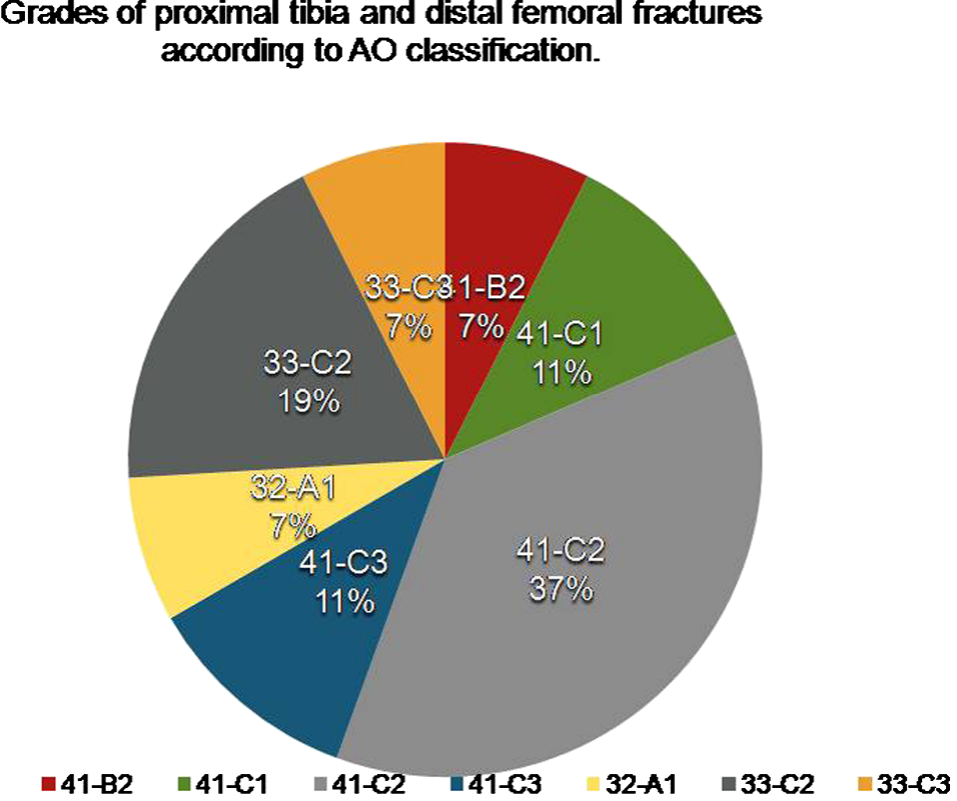
Types of tibial plateau and distal femoral fractures included in this study classified according to Muller's AO classification system.

Twenty seven patients with acute intra and peri-articular fractures around arthritic knees had TKA ± internal fixation as a primary and definitive treatment. Nine of these fractures were distal femoral fractures and the remaining 18 were fractures of the tibial plateau (Figure 1). The mean age of these patients was 63 years (range 59–74 years).

This is a single surgeon* cohort of patients who were enrolled in the protocol of management and prospectively evaluated in the period between June 2008 to July 2014.

Fractures of the proximal tibia and distal femur were classified according to Muller's AO classification system [10].

Intra-articular fractures with haemoarthrosis were initially immobilized in a knee brace for 4 weeks. This period allowed absorption of the hematoma, reduction of the swelling, inflammation and bruises of the soft tissue prior to the surgical intervention. Low Molecular Weight Heparin as a chemical prophylaxis against thromboembolic complications was prescribed in the appropriate dose for every patient during the period of immobilization.

**Figure 2:**
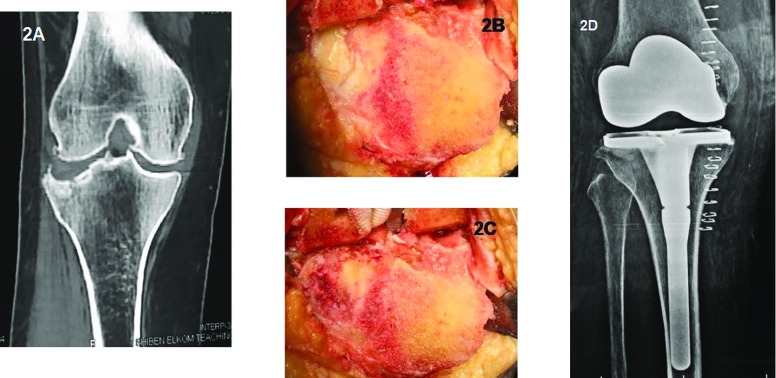
(A) Preoperative CT scan showing lateral depression of the tibial plateau and medial joint arthritic changes. (B) Intra-articular photo after proximal tibial cut. Lateral depressed articular surface with remaining cartilage covering. (C) Peripheral bone defect after removal of the depressed articular cartilage. (D) Postoperative X-ray with a stem added to the tibial tray and cement filling of the lateral defect.

The following technical steps were followed during the surgical procedure:

Standard midline incision with medial para-patellar approach was performed in all cases. For depressed tibial plateau fractures, no elevation of depressed metaphyseal fragments was attempted. Minimal bone resection was performed using extra-medullary alignment jigs that was referenced to the less affected side of the arthriticsurface. Articular cartilage covering depressed articular fragments was removed using bone curet leaving peripheral or contained metaphysical defects (Figure 2).

**Figure 3:**
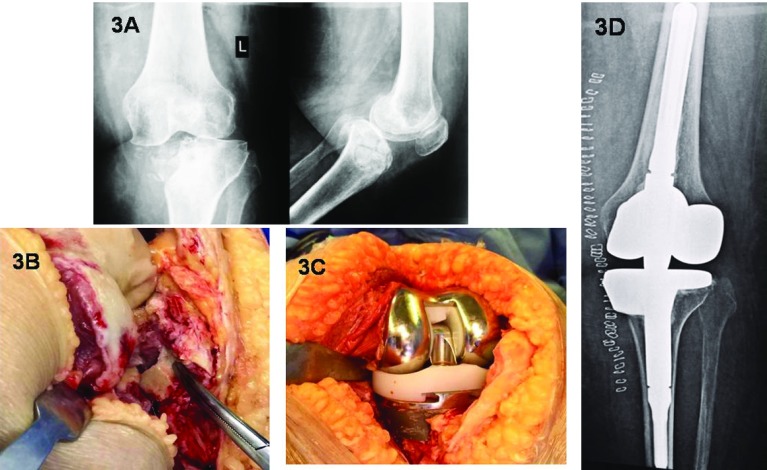
(A) Preoperative X-rays for a depressed fracture of the medial tibial plateau. Metaphyseal collapse and knee subluxation can be observed. (B) Intraoperative photo shows unreconstructable comminution of the articular surface. (C) The bone defect was reconstructed using a medial 20 mm metal step attached to the tibial tray. (D) Postoperative X-ray showing the Rotating Hinge implant and metal augment to the proximal tibia.

Large articular fragments that were highly comminuted and unstable were discarded. Defects in the proximal tibia were then reconstructed using metal augments added to the tibial tray (Figure 3). When the Tibial Tubercle (TT) was unstable trans-osseous sutures were performed to stabilize the TT at the end of the procedure.

An extension stem 145 mm in length was added to the tibial tray (Zimmer Inc, Warsaw, Indiana) in all cases of tibial plateau fractures even when a primary Posterior Stabilized (PS) polyethylene insert was chosen (Figure 2D).

**Figure 4:**
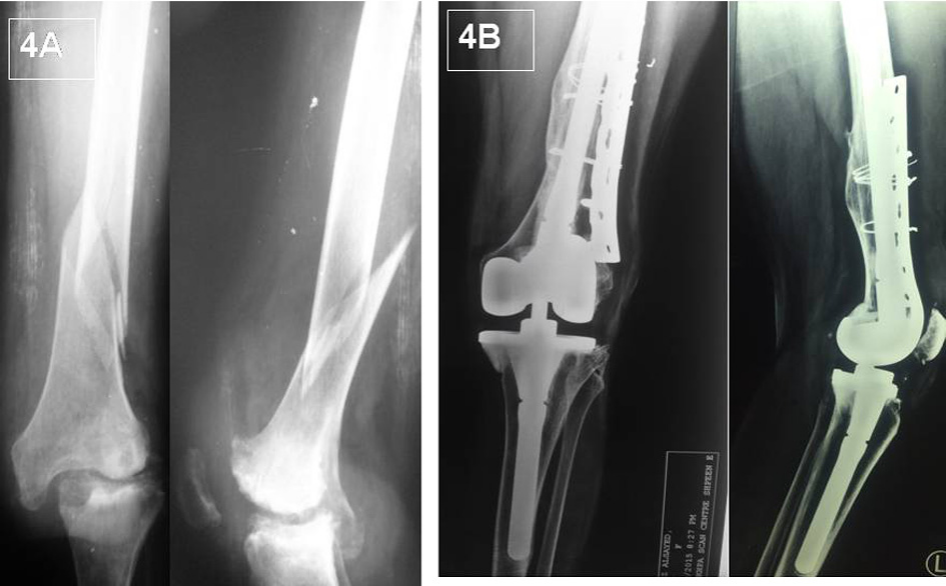
(A) Preoperative X-rays for a 63 rheumatoid lady with advanced knee arthritis and acute distal femoral fracture. (B) The strategy of fix and replace was followed with simultaneous fixation of the fracture and replacement of the knee using an LCCK implant. 7 years follow up with full union of the fracture and stable functioning knee prothesis.

For distal femoral fractures, the procedure started by fixing the fracture and restoring the position of the femoral epicondyles and ligament attachments. This was then followed by performing the femoral cuts using intra-medullary alignment rod and adding a stem to the femoral component. The stem length was chosen to bypass the fracture site by what is equivalent to two widths of the medullary canal. Legacy Constrained Condylar Knee (LCCK) femoral component and insert was chosen for intra-articular femoral fractures (Figure 4). However, when comminution of the articular surface involved the collateral ligaments attachment and adequate balancing of flexion-extension gaps could not be achieved a Rotating Hinge (RH) prosthesis was chosen.

Hybrid form of fixation was performed on the tibial and femoral sides of the prostheses. Medium viscosity antibiotic loaded bone cement covered the surface of the tibial trays and femoral components while the stems were non-cemented (Figure 2).

PS polyethylene insert was used when accurate ligament balancing was achieved which was the case in the majority of tibial plateau fractures. Alternatively, a semi-constrained LCCK or a RH implants were chosen to overcome global ligament insufficiency following a knee dislocation or total loss of the Medial Collateral Ligament (MCL) function.

Patellar resurfacing was performed when advanced erosion of its articular surface was observed or in cases of rheumatoid arthritis.

One patient in this series with a BMI of >40 had highly comminuted distal femoral fracture that was not possible to reconstruct. Hence a distal femoral replacement prosthesis (LINK) was chosen and cemented stems were employed on the femoral and tibial sides.

All cases were allowed early weight bearing as tolerated from the second post-operative day. A hinged knee splint was used during weight bearing until full quadriceps control was regained. In cases of combined arthroplasty and fixation of distal femoral fractures, the hinged brace was used for 6 weeks post-operatively.

A protocol of multimodal postoperative analgesia including epidural catheter was used for 3–4 days postoperatively and chemical prophylaxis for thromboembolism was continued for 4 weeks.

Radiological evaluation was carried out at 3, 6, 12 months post operatively and then annually thereafter. Clinical evaluation of the quadriceps function, knee ROM and clinical Knee Society Scores (KSS) were recorded [11].

## Results

Twenty seven patients with an average age of 63 years (range 59–74) were prospectively evaluated. Twenty five of these patients were females while only two were males. The average follow up of these patients was 6 (range 3–8) years. At the latest follow up two of these patients had ceased from causes unrelated to their knee procedures leaving 25 patients for the final evaluation.

**Figure 5:**
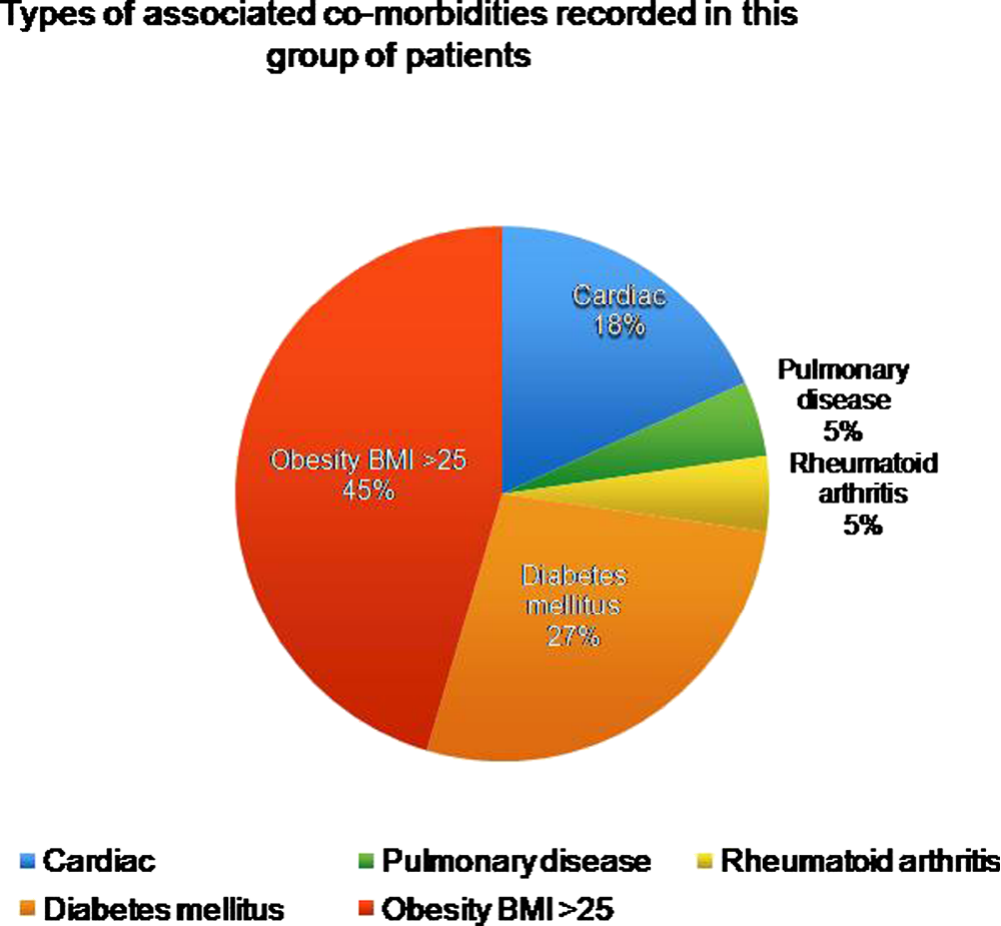
Percentage of various general health problems and co-morbidities found in this cohort of patients.

This was a group of elderly patients with various degrees of general health problems as summarized in Figure 5.

Eighteen of these fractures were tibial plateau fractures that needed reconstruction of the tibial side and a stem extension added to the tibial tray. Restoration of the joint line and accurate ligament balancing was achieved in 16/18 and consequently a standard PS insert was employed. In cases of distal femoral fractures, the LCCK was implanted in 8 patients while a Nex-Gen RH implants were used in two knees. Distal femoral replacement prosthesis (LINK) was necessary to use in only one patient with highly comminuted distal femoral fracture and severe osteoporosis.

At the latest follow up the average KSS for the operated knees was 80 (range 75–89) points. All patients achieved full knee extension and good range of knee flexion that was 110° on average (between 90° and 135°). All patients achieved a high score in the pain item of the KSS ≥40 points.

Radiological evaluation revealed full bony union of all fractures. All knee protheses were radiologically stable with no radiolucent lines observed around the tibial trays or femoral components except one tibial stem.

One patient had early post operative chest infection with type II respiratory failure. The same patient had continued wound discharge and needed DAIR. Antibiotics were infused for 6 weeks after the debridement according to culture and sensitivity results and no further intervention was needed.

## Discussion

The most important finding from this study was that TKR as a primary treatment for knee fractures in elderly population achieves excellent results comparable to primary TKR for arthritis. These results were maintained at the medium term follow up of average 6 years.

Comminuted intra-articular fragility fractures in the elderly have been a challenge to the orthopedic surgeons. This is especially true when arthritis preexisted. Failure of fixation, rapid progression of arthritis and stiffness are usually contributing to the unsatisfactory results of internal fixation to this particular group of patients [12,13].

Though TKR is a highly successful procedure for the treatment of knee arthritis, when performed as a delayed procedure to salvage a malunited or non-united tibial plateau fractures it was associated with high incidence of stiffness, infection and disruption of the extensor mechanism [4–6,14,15]. Therefore, distal femoral replacement was suggested as a primary procedure for the highly comminuted fractures [7–9].

A report on the 1-year mortality following low energy distal femoral fractures in elderly patients correlated between the delay in the surgical intervention and an increased rate of postoperative mortality. This finding was, however, on patients with high Charlson Comorbidity Score indicating poor general health status that lengthened the time of preoperative optimization and postoperative hospital stay [16].

If TKR is to be performed as a primary procedure to treat intra-articular fractures in the elderly, various issues come into question. The first are technical questions related to the timing of the surgical intervention, choice of implant and post operative protocol. Secondly, how successful is the procedure in terms of post operative function and ROM. Finally, will the revision implants used in this particular group of patients have higher revision rates when compared to a primary TKR for arthritis?

Integrity of the soft tissue envelope around the knee is an essential element to achieve satisfactory outcome in cases of TKA. Though knee fractures in elderly patients are usually the result of low energy type of trauma they are still associated with knee hemoarthrosis, swelling and ecchymosis. Therefore, splinting of the knee for four weeks allows for reduction of soft tissue swelling and inflammation before proceeding to the knee replacement. The interval between the time of the fracture and operative intervention could be adjusted in every case depending on the level of the fracture, the degree of swelling and skin condition. In general, tibial plateau fractures need longer periods for the swelling to resolve and skin condition to improve when compared to distal femoral fractures.

In type 41-B3 depressed lateral tibial plateau fractures, the proximal tibial cut was performed by referencing to the intact medial tibial articular surface. Depressed metaphyseal fragments have usually created peripheral or contained bone defect <10 mm in depth (Figure 2). These defects can simply be filled by bone cement and the addition of a stem to the tibial tray [6].

Meanwhile, significant bone defects at the articular surface found in type 41-C1 tibial plateau fractures needed metal augments to support the tibial tray. Therefore, the stability of the tibial trays were achieved by a combination of proximal bone support and intramedullary diaphysial stem contact. As the majority of the tibial plateau fractures were low energy fractures in osteoporotic bone, these techniques were adequate to achieve primary stability and allow early weight bearing.

The TT was part of the fracture in two cases. Attachment to the metaphyseal bed using three doubled trans-osseus sutures of Ethibond no. 5 secured the TT in position.

Appleton et al. [17] presented the 10 years results of knee arthroplasty as a primary treatment for distal femoral fractures. The authors reported 42% incidence of mortality within the first postoperative year from associated cardiac and pulmonary co-morbidities. There were, however, few early postoperative complications infection and hematoma formation. In addition, they reported 4 early periprosthetic fractures between the end of the knee femoral stem and ipsilateral hip implants [17]. Presence of a stress riser in the area that separates between knee and hip implants should always be considered in the preoperative planning of stemmed knee implants when there is a proximal hip prothesis or metalwork. Total femoral replacement was recently suggested to avoid future periprosthetic fractures.

It is important here to highlight the fact that Appleton's [17] cohort of patients were much older (average age 82 years), of very limited preoperative mobility and finally their policy of management in the acute trauma ward setting using a distal femoral replacement prothesis is different from the reported strategy in this article for delayed (2–4 weeks after trauma) joint arthroplasty in arthritic knees with periarticular fracture.

Similar to our strategy, Choi et al. [8] reported a series of distal femoral fractures that was treated with the medial pivot knee employing a similar strategy of fixing simple distal fractures by wires and screws and a stem added to the femoral component. The authors reported good outcome in terms of fracture healing and functional ROM [8].

In this series initial fixation of distal femoral fractures by lateral plate in addition to uni-cortical screws distally and circular wires proximally provided the necessary support for performing the bone cuts using an intra-medullary guide. This technique of reconstruction allowed using less constrained form of prosthesis. However, highly comminuted fractures that involved the articular surface required the use of RH implants in 2 knees and even a distal femoral replacement prosthesis in one. Stiffness and patellar tendon avulsion are common complications when TKR is performed as a salvage for malunited tibial plateau fractures. In this scenario, knees have extra-articular adhesions that negatively affects the postoperative ROM. Additionally, non weight bearing increases the osteoporosis especially at sites of tendinous insertion into the bone. Hence, the high rate of these two particular complications can be explained in previously published studies [5,6].

In this series the post-operative ROM was comparable to primary TKR for arthritis with flexion to average 110° (range 90–135°) and full extension to zero in all cases.

These good results can be explained by the early intervention and post operative protocol of exercises and weight bearing. An essential target to be achieved is to have a stable construct by the end of the procedure. Consequently, fast rehabilitation and weight bearing can be tolerated without waiting for the fractures to heal.

This procedure is usually suggested for patients with marked osteoporosis. Non-weight bearing would worsen their bone density. Therefore, the benefit from this surgery is not only related to improving knee function but may also be reflected on their bone quality and the degree of osteoporosis that could be looked at in future research.

An area of concern to the knee surgeon would be how much bone preserving is this procedure and how easy a future revision will be if needed? The procedure is suggested for elderly patients in their sixties or seventies and allows early weight bearing that is likely to be positively reflected on these patients general well being and the quality of their lives. Therefore, if TKR in this particular group of patients achieves excellent long term survival as seen with the mid term, the question becomes then irrelevant.

Though this study included a small group of patients and reflects a single surgeon's experience; it is one of the largest series to be published on this particular indication and the first to present medium term outcome without loss to follow up.

Finally, when performed for the suitable candidates having optimized their soft tissue condition; TKA provides excellent results as a primary treatment for knee fractures in the elderly population at the medium term.

## Conflict of interest

The authors declare that they have no conflict of interest.

## Funding

There is no funding source.

## Ethical approval

Informed consent was obtained from all individual participants included in the study.
